# Optimization of loop-mediated isothermal amplification (LAMP) assays for the detection of *Leishmania* DNA in human blood samples

**DOI:** 10.1016/j.actatropica.2016.06.009

**Published:** 2016-10

**Authors:** Ibrahim Abbasi, Oscar D. Kirstein, Asrat Hailu, Alon Warburg

**Affiliations:** a Department of Microbiology and Molecular Genetics, The Institute for Medical Research Israel-Canada, The Kuvin Centre for the Study of Infectious and Tropical Diseases, The Hebrew University – Hadassah Medical School, The Hebrew University of Jerusalem, 91120, Israel; b Department of Biological Sciences, Faculty of Science and Technology, Al-Quds University, Abu Deis, the Palestinian Territories; c Department of Microbiology, Immunology & Parasitology, Faculty of Medicine, Addis Ababa University, Addis Ababa, Ethiopia

**Keywords:** Isothermal loop-mediated amplification, SYTO16, Real time LAMP, Visceral leishmaniasis, *Leishmania donovani*

## Abstract

Visceral leishmaniasis (VL), one of the most important neglected tropical diseases, is caused by *Leishmania donovani* eukaryotic protozoan parasite of the genus *Leishmania,* the disease is prevalent mainly in the Indian sub-continent, East Africa and Brazil. VL can be diagnosed by PCR amplifying ITS1 and/or kDNA genes. The current study involved the optimization of Loop-mediated isothermal amplification (LAMP) for the detection of *Leishmania* DNA in human blood or tissue samples. Three LAMP systems were developed; in two of those the primers were designed based on shared regions of the ITS1 gene among different *Leishmania* species, while the primers for the third LAMP system were derived from a newly identified repeated region in the *Leishmania* genome. The LAMP tests were shown to be sufficiently sensitive to detect 0.1 pg of DNA from most *Leishmania* species. The green nucleic acid stain SYTO16, was used here for the first time to allow real-time monitoring of LAMP amplification. The advantage of real time-LAMP using SYTO 16 over end-point LAMP product detection is discussed. The efficacy of the real time-LAMP tests for detecting *Leishmania* DNA in dried blood samples from volunteers living in endemic areas, was compared with that of qRT-kDNA PCR.

## Highlights

• Three systems of loop-mediated isothermal amplification (LAMP) were developed for diagnosing leishmaniasis.
• The green nucleic acid stain, SYTO-16 was adapted for monitoring the reactions in real-time.
• The LAMP assays proved highly sensitive detecting >100Fg DNA/reaction.
• *Leishmania* DNA was detected in a significant number of asymptomatic individuals living in endemic areas.

## Graphical abstract

**Figure:**
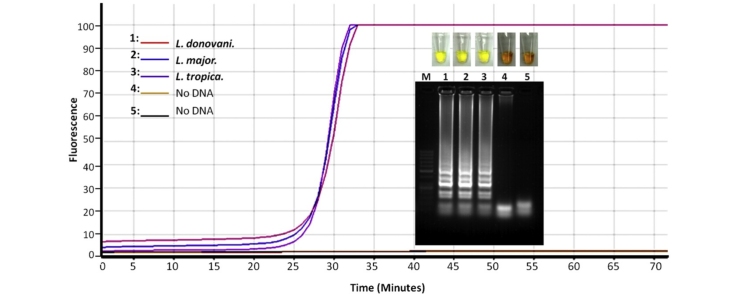

## 1 Introduction

The leishmaniasis are a group of diseases, caused by eukaryotic protozoan parasites of the genus *Leishmania,* transmitted by blood-sucking phlebotomine sand flies. Leishmaniasis affects 12 million men, women and children in 88 countries around the world, over 350 million humans are at risk, and over 2 million new cases emerge every year (Alvar et al., 2012, Ready, 2014, WHO/TDR, 2010). Visceral leishmaniasis (VL), the most serious form of the disease, occurs mainly in the Indian sub-continent, East Africa and Brazil. Although some 400,000 people are affected by VL annually with up to 40,000 deaths per year, the disease is still ranked among the neglected tropical diseases (Alvar et al., 2012, Desjeux, 2004, Ready, 2014).

VL can be diagnosed by microscopic examination of stained, splenic or bone marrow biopsy material (Chappuis et al., 2007, WHO/TDR, 2010). Serological methods with moderate sensitivity based on direct agglutination test (DAT) and enzyme-linked immune-sorbent assays (ELISA) are also used (Boelaert et al., 2008, ter Horst et al., 2009). In addition, several other methods based on *in vitro* DNA amplification of different repeated *Leishmania* genes, using the polymerase chain reaction (PCR), were also adapted for detection of parasites in tissue samples. The most commonly targeted genes are the ribosomal ITS1 and the kinetoplast minicircle genes (kDNA) (el Tai et al., 2000, Nicolas et al., 2002, Rodgers et al., 1990, van Eys et al., 1992). These PCR assays have been developed for the detection of *Leishmania* DNA in a variety of clinical samples such as skin biopsies and smears, bone marrow and lymph node aspirates as well as peripheral blood. To date, there is no generally-accepted gold standard for *Leishmania* identification (Miller et al., 2014). Van der Auwera and Dujardin (2015), gave a comprehensive literature analysis for the different available and accepted methods used for *Leishmania* species detection in clinical and epidemiological studies.

Rapid, minimally invasive diagnostic test for cutaneous leishmaniasis (CL) would constitute a valuable tool, especially when large numbers of patients are to be tested in a short time and under field conditions. In the case of VL, prevalent asymptomatic infections in endemic regions may serve as parasite reservoirs for infecting vectors (Adler et al., 1966, Ali and Ashford, 1994, Sharma et al., 2000). Thus, a rapid and simple field-test to determine putative reservoir potential of *L. donovani*, would comprise a valuable tool for curtailing disease transmission.

Loop-mediated isothermal amplification (LAMP) was first introduced by Notomi et al. in 2000. The technique is considered a highly sensitive and rapid method for DNA amplification at a constant temperature (60–65 °C). LAMP can amplify a few copies of DNA to 10^9^ molecules in about one hour under isothermal conditions The methodology relies on auto-cycling strand-displacement DNA synthesis using *Bacillus stearothermophilus* (Bst) DNA polymerase, and the use of 4 specially designed primers that enable the production of amplification products having a stem and loop structure. LAMP has proved its usefulness for the detection of many infectious agents including disease–causing parasites (Abbasi et al., 2013, Aryan et al., 2010, Notomi et al., 2000, Poon et al., 2006, Salant et al., 2012).In recent years several studies have utilized LAMP for detecting *Leishmania* DNA in tissue and blood of infected humans as well as the sand fly vectors (Khan et al., 2012, Nzelu et al., 2014, Takagi et al., 2009, Verma et al., 2013). The first LAMP test for diagnosis of infection was developed by Takagi et al., who used specific primers for *L. donovani* that were based on kDNA sequences and achieved an amplification sensitivity of 1fg per reaction (Takagi et al., 2009). Later on, this test was used by other researchers to validate VL infections from patients' blood samples (Khan et al., 2012, Verma et al., 2013).

We designed three sets of primers for LAMP DNA amplification appropriate for the detection of most *Leishmania* species. Two sets of primers derive from shared regions of the *Leishmania* ITS1 sequences and the third set was obtained from a newly-identified repeat region known to have shared sequences in *L. donovani* and *L. major*. Results showed that the sequence is also shared by other *Leishmania* species. The described method employs SYTO-16, a nucleic acid stain found to be suitable for real-time LAMP detection by fluorescence. Thus, our assay avoids the necessity for end-point analysis, be it using green fluorescence (SYBR Green I), turbidity (spectrophotometry measurement of absorbance at 400 nm) or agarose gel electrophoresis.

## 2 Materials and methods

### 2.1 Ethical concerns

Informed consent was sought from all the adults recruited for the study. Consent for inclusion of young children, was obtained from parents or guardians. Study procedures were approved by the ethical review committees at the Medical Faculty, Addis Ababa University and the National Research Ethics Review Committee (NRERC) at the Ethiopian Ministry of Science and Technology.

### 2.2 Samples

Genomic DNA was obtained from the following *Leishmania* reference strains: *L. donovani* (MHOM/SD/62/IS), *L. major* (MHOM/IL/2014/LRC-L1671), *L. tropica* (MHOM/IL/2011/LRC-L1558), *L. aethiopica* (MHOM/ET/72/L100), *L. infantum chagasi* (MHOM/CR/199?/LRC-L744), *L. mexicana* (MNYC/BZ/62/M 379), *L. amazonensis* (MHOM/BR/73/M2269), *L. braziliensis* (MHOM/BR/75/M-4037), *L. panamensis* (MHOM/CO/86/UA126), *L. b. guyanensis* (MHOM/BR/75/M4147). Human finger-prick blood samples were collected from 44 individuals living in a village in the Tahatay Adiabo district of north Ethiopia. From these 44 samples; 9 individuals were previously treated VL cases residing in different households. The rest of the samples were taken from volunteers living nearby.

### 2.3 DNA extraction

Total genomic DNA was purified using phenol-based DNA extraction method (28). *Leishmania* promastigotes or ∼20 μl dried blood (blotted in two 6 mm [dia.] 3MM filter paper punches) were incubated at 60 °C for two hours in a 1.5 ml micro-centrifuge tube containing 200 μl of lysis buffer (50 mM NaCl, 10 mM EDTA, 50 mM Tris-HCl pH 7.4, 1% triton X-100, and 200 μg/ml of proteinase K). This was followed by extraction with equal volumes of TE-saturated phenol (pH 8), and then precipitating the DNA with ethanol. The extracted lyophilized DNA was resuspended in 50 μl DNAse/RNase-free, double distilled water and stored at −20 °C until further use.

### 2.4 LAMP primer design

**Fig. 1:**
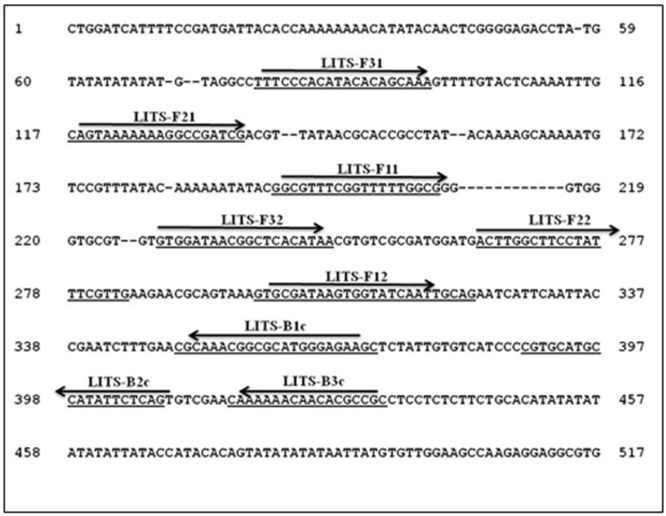
DNA sequence of the *L. donovani*ITS1 gene, showing the location of the LAMP primers on the sequences shared by different *Leishmania* species.

Three different sets of LAMP primers (external primers: F3 and B3, internal primers: FIP and BIP) were designed; the sequences for two sets were based on shared ITS1 DNA sequences of different *Leishmania* species. The third set of primers was based on *L. donovani* repetitive sequence as elaborated in the “results” section. All primers were designed manually taking into consideration the required melting temperatures, and the distances between all the primers. Table 1 shows the nucleotide sequences of the newly designed LAMP primers, and Fig. 1 presents their location within the conserved *Leishmania* ITS1 gene.

### 2.5 LAMP assays

LAMP reactions were performed in 25 μl enzyme reaction buffer (20 mM Tris-HCl [pH 8.8], 10 mM KCl, 10 mM [NH_4_]_2_ SO_4_, 8 mM MgSO_4_, and 1% Tween 20).; that contains 40pmoles FIP and BIP; 5pmoles F3 and B3 outer primers; 8 units of Bst-WarmStart DNA polymerase (New England Bio-labs Inc., MA, USA);0.2 mM dNTPs mixture; 0.8 M Betaine; 2 μM SYTO-16 green fluorescence nucleic acid stain (Thermo-Fisher Scientific, Grand Island, NY).The reaction was carried out at 65 °C for 2 h in a real time PCR thermocycler (Rotor-Gene 6000, Qiagene, Hilden, Germany) with fluorescence data acquisition at one minute intervals.

### 2.6 Detection of LAMP

**Fig. 2:**
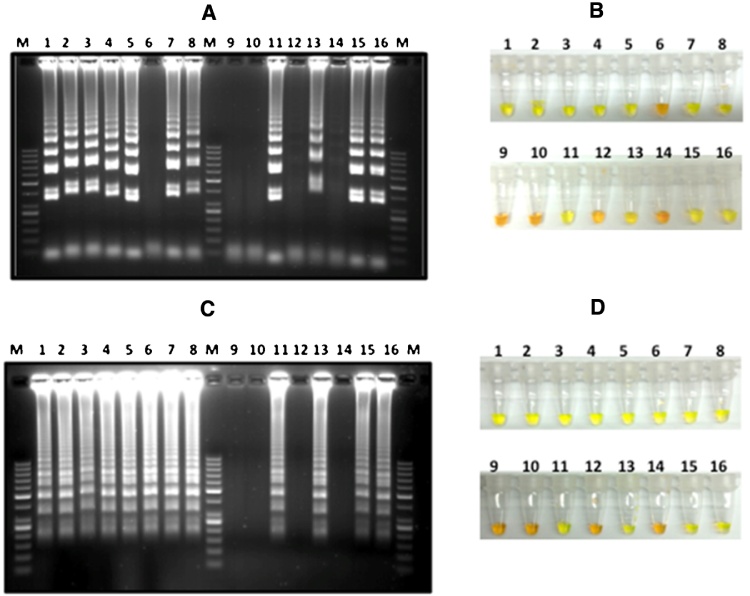
Agarose gel electrophoresis analysis and SYBR Green I end point detection of LITS-LAMP1 (A,B), and LITS-LAMP2 (C,D). The analysis shows LAMP DNA amplification products of different concentrations amplified from different amounts of *L. donovani* template DNA, (1) 0.1 ng, (2) 0.01 ng, (3) 1 pg, (4) 0.1 pg. And the amplification of 1 ng template DNA of each of the following *Leishmania* species: (5) *L. major*, (6) *L. aethiopica*, (7) *L. tropica*, (8) *L. infantum chagasi*. (9) and (10) No DNA [control], (11–16) Select blood samples obtained by finger prick from volunteers in North Ethiopia. (M) DNA size marker.

DNA amplification was assessed using two methods. The SYTO^®^16 green-fluorescent nucleic acid stains are cell-permeant nucleic acid stains that show intense fluorescence enhancement upon binding nucleic acids. SYTO-16 (2 μM, Excitation/Emission, 488/518 nM) was used for real-time detection and quantitation of the DNA accumulating in the reaction tubes.The second method employed end-point quantification of DNA by adding SYBR Green-I(>1 μM,Thermo-Fisher Scientific, Grand Island, NY). SYBR Green-I detects the presence of LAMP-DNA immediately causing the color of the reaction to change from orange to green (Fig. 2). To verify the results, LAMP–DNA was electrophoresed and viewed on standard agarose gels stained with ethidium-bromide (Fig. 2).

### 2.7 Quantitative real-time kinetoplast DNA PCR (qRT-kDNA PCR)

The concentration of *Leishmania* parasites in blood was estimated as described previously (Abbasi et al., 2013). Briefly, kDNA amplification by qPCR was achieved usingt he kDNA minicircle-specific primers JW11 (CCTATTTTACACCAACCCCCAGT) and JW12 (GGGTAGGGGCGTTCTGCGAAA) (Nicolas et al., 2002). The qPCR reaction was performed using the Absolute Blue qPCR SYBR Green mix kit (Thermo scientific, Surrey, UK), and the amplified DNA was detected and quantified by a real time PCR thermocycler (Rotor-Gene 6000, Qiagene, Hilden, Germany).

## 3 Results

### 3.1 Selection of *Leishmania* LAMP primers

ITS1 Sequences from different *Leishmania* species selected from GenBank, were aligned in order to identify the DNA sequences shared by all *Leishmania* species and, thus, suitable for LAMP primers. Based on comprehensive ITS1 alignments; two external forward (F3) primers (LITSF3.1 and LITSF3.2), and one backward (LITSB3) primer were identified. In addition, two forward internal primers(LITSFIP1 and LITSFIP2) and one backward inner primer (LITSBIP) were designed. These primers were used to build two *Leishmania* ITS1LAMP systems (LITS-LAMP1 and LITS-LAMP2). Where; LITS-LAMP1 spans 353 bp, and LITS-LAMP2 covers 204 bp (Table 1, Fig. 1).

The selected primers for the third *Leishmania* LAMP system (L151) (Table 1) were based on a *L. donovani* repetitive DNA sequence (458-bp) identified previously (Abbasi, unpublished data). The repeat sequence forms part of conserved *L. donovani* gene segment (GenBank accession no. XM-003858114), and it also displays a high degree of homology to a segment in the *L. major* chromosome 4 (GenBank accession no. FR796400), but no known homology with other *Leishmania* species.

### 3.2 Optimization of the detection of real-time LAMP DNA amplification

**Fig. 3:**
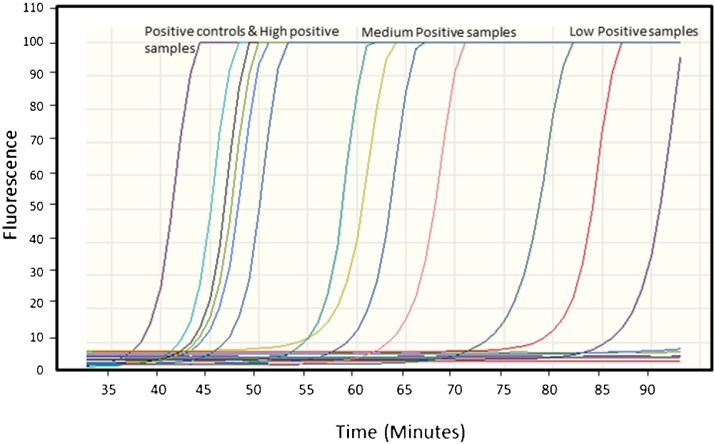
Monitoring of LAMP amplification from different *Leishmania* DNA control samples and from DNA extracted finger pricks samples. The analysis shows the DNA amplification detection in real time by measuring the increasing fluorescence of DNA binding to SYTO-16 dye.

A comparison between SYBR Green I and SYTO-16 fluorescent nucleic acid showed that SYBR Green-I at concentrations of 1–5 μM inhibited LAMP amplification almost completely. Real-time detection of DNA amplification was possible only at concentrations below 1 μM. However, at these extremely low concentrations, fluorescence intensity was lower than 50% of the intensity required for detection (data not shown) severely limiting its efficacy. On the other hand, SYTO-16 (2 μM) allowed optimal real time visualization of the LAMP products. Fig. 3, shows real time detection of LAMP DNA amplification detected on a qRT-PCR thermocycler using SYTO-16.

### 3.3 LAMP sensitivity and specificity

LAMP was performed using different concentrations of *L. donovani* template DNA (0.1 ng, 0.01 ng, 1 pg, and 100fg) and 1 ng DNA from additional *Leishmania* species from the Old World and from Latin America (*L. major, L. tropica, L. aethiopica*, *L.* [*infantum*] *chagasi, L. mexicana*, *L. amazonensis*, *L. braziliensis*, *L. panamensis*, *and L. guyanensis*). LITS-LAMP1/2 and L151, amplified *L. donovani* DNA at the lowest concentration tested (100fg, Fig. 2), similar levels of sensitivity were also obtained with *L. major* template DNA. While LITS-LAMP2 and L151 amplified DNA from all the *Leishmania* species tested, LITS-LAMP1 failed to amplify DNA from *L. aethiopica* (Fig. 2).

### 3.4 Samples analysis

The three LAMP systems were compared for their ability to detect *Leishmania* DNA in finger prick blood collected from 44 individuals living in a VL endemic area in Ethiopia. LAMP results were compared with those obtained by qRT-kDNA PCR. All volunteers were asymptomatic at the time of sampling although some had succumbed to VL and were treated in the past (Table 2, column 2). Two samples (number 22 and 36) showed positive results with all the three LAMP systems as well as the qRT-kDNA PCR. The strength of LAMP positivity was indicated by the time (in minutes) needed for obtaining fluorescence signals caused by the binding of SYTO-16 to the amplified DNA. This parameter is known as take-off time, and the shorter time means relatively more template DNA in the sample. The take-off times of LITS-LAMP1, LITS-LAMP2, and L151-LAMP using 0.1 ng *L. donovani* template DNA were 42, 40, and 54 min respectively. Takeoff times longer than 95 min for LITS-LAMP1 and LITS-LAMP2, and 110 min for L151-LAMP were considered negative. Note sample number 22; that showed the highest parasite number by kDNA-qPCR, also showed an early take-off time in the three LAMP systems (Fig. 3).

Of the nine previously-treated VL cases; five and six individuals were positive by LITS-LAMP2 and L151 LAMP respectively. Only three of those were positive by qRT-kDNA PCR. Of the nine samples positive by qRT-kDNA PCR, three, seven and 5 were also positive by LITS-LAMP1, LITS-LAMP2, and L151 LAMP respectively (Table 3). Looking at the results of the shared positives between kDNA-PCR and LITS-LAMP2 (that was 7/9), the other two samples that were negative by LITS-LAMP2 were found to have 3, and 9 parasites/ml, based on previous validation of kDNA-PCR results it was calculated that 41.7% of the estimated parasite number with less than 10 parasites/ml could be false positives (Abbasi et al., 2013).The LAMP system that detected the highest number of positive individuals was LITS-LAMP2 (19 positives from 44) followed by L151-LAMP that detected 13 including 10 positive individuals whom were also detected by LITS-LAMP2 (Table 3).

## 4 Discussion

In this study we evaluated the efficacy of several newly developed LAMP assays for detecting low concentrations of *Leishmania* DNA in biological samples. The main advantages of LAMP over conventional and qRT-PCR, are the extremely high sensitivity (detection of 1fg target DNA per reaction (Aryan et al., 2010, Notomi et al., 2000)), the speed with which the assays can be completed, and the simple, relatively inexpensive equipment required. These traits make LAMP assays readily adaptable to field conditions (Hamburger et al., 2013, Njiru, 2012). The three LAMP systems we describe here, detected very low concentrations (>100fg) of *L. donovani* DNA. This is about 10^4^ more sensitive than conventional ITS1-PCR (el Tai et al., 2000), and about 10^3^ more sensitive than kDNA-PCR (Nicolas et al., 2002). While high sensitivity is certainly an asset, it can also be a liability, necessitating special care to avoid contamination during DNA preparation and mixing of the reaction components in order to avoid false positive results. Thus, it is preferable not to open the tubes at the end of the LAMP reaction for endpoint analysis by SYBR Green I or agarose gel electrophoresis (Kubota and Jenkins, 2015). This can be achieved by adding assimilating primers; one fluorescent and one quenching to the reaction mixture. These primers fluoresce only once they are assimilated into the LAMP product, thereby, demonstrating a positive LAMP reaction (Kubota and Jenkins, 2015).

We selected a direct nucleic acid stain (SYTO-16) to follow the LAMP reactions in real time and avoid the necessity to open the reaction tubes. Selection of a suitable fluorescent dye to be used in real time LAMP detection is crucial (Watts et al., 2014). Previous studies clearly showed that SYBR green I, the most commonly used DNA fluorescent dye, inhibits LAMP reactions (Salant, 2013). The use of 1 μM SYTO-16 dye enabled real time monitoring of LAMP at a low concentration with no inhibitory effect. The importance of developing a LAMP test is its use for point of care nucleic acid based diagnosis, so the advantage of incorporating such low concentration of SYTO-16 dye, renders such tests inexpensive, beside it is possible to use a portable real time machine for monitoring the LAMP amplification (Kubota and Jenkins, 2015, Seyrig et al., 2015).

The shortest takeoff times (35–45 min) were those of the positive controls (Fig. 3). The positive test samples took-off during some 30 min thereafter, while any signals that appeared after 95 min were considered false positives since, at that stage, many of the negative controls with no target DNA or known non-endemic negative human DNA also started to fluoresce. In fact, real time monitoring showed that LAMP reactions produced false positive signals if allowed to proceed for 95 min or longer (data not shown). Similarly, in conventional PCR, amplification occurring after 35 cycles is frequently attributable to spurious priming and considered false positive. Thus, when performing endpoint analyses, LAMP reactions need to be calibrated for maximum incubation with minimal false positive results by running positive control samples with ascending concentrations of template DNA, for different lengths of time before adding SYBR Green I. This illustrates the main advantage of real time LAMP using SYTO-16 over end-point evaluation.

Previous studies using *L. donovani*-specific LAMP assays showed a sensitivity of 90.7% in one study (Khan et al., 2012), and 96.4% in another study using the same LAMP system. Both studies were performed on samples from clinically-confirmed VL cases (Verma et al., 2013). The diagnostic LAMP assays described previously were based on a primer sets designed specifically for the detection of low concentrations (1fg) of kinetoplast mini-circle DNA of *L. donovani* in blood and tissue samples (Takagi et al., 2009, Verma et al., 2013). Another LAMP amplification system was developed based on the *Leishmania* 18S rRNA gene for the detection of *Leishmania* DNA in cutaneous lesions tissue spotted on FTA card. Malachite green was used for end point detection (Chaouch et al., 2013). The use of a reverse transcriptase LAMP assay based on the 18S ribosomal RNA for the diagnosis of visceral leishmaniasis and cutaneous leishmaniasis was also possible with a less sensitivity than direct detection of DNA by LAMP assay (Adams et al., 2010). In contrast with the previously developed LAMP tests, the LAMP assays we describe were designed to amplify DNA from as many *Leishmania* species as possible. Once adapted to run on a field LAMP apparatus, our general LAMP assays may be useful for epidemiological field surveys rather than for diagnostic purposes, even when more than one species of *Leishmania* species is endemic (Hamburger et al., 2013, Njiru, 2012).

In VL foci, a large proportion of asymptomatic persons are frequently infected with *L. donovani* or *L. infantum* (Abbasi et al., 2013, Topno et al., 2010, Ukil et al., 2011). For example in an endemic focus in Bihar, India, the rate of sero-conversion among healthy individuals residing in households with a history of VL was 62%, and the disease conversion rate in PCR positive subjects was higher than sero- positivity (Ukil et al., 2011). In another epidemiological study from Nepal, Anti-*Leishmania* antibodies were found in 40/416 (9.6%) persons without VL and the risk factor for VL upon having a VL case in the neighbourhood was higher (Ostyn et al., 2015), the same group were able to detect *Leishmania* DNA in 5% of the individuals using *Leishmania* SSU-rDNA PCR. We focused on, asymptomatic volunteers; nine of whom were previously-treated VL cases. The numbers of previously-treated cases shown to be infected were 9/44 by qRT-kDNA PCR, 6/44 by LITS-LAMP1, 19/44 by LITS-LAMP2, and 13/44 by L151-LAMP. Thus, the LAMP tests indicate the presence of parasite DNA in the blood of some asymptomatic individuals. Identifying asymptomatic cases based on immunological or molecular analysis among household members of individuals with previously confirmed VL cases can classify this group as being at risk of infection. In different studies the use of molecular methods for *Leishmania* detection identified higher percentages of asymptomatic cases (Alborzi et al., 2008). In addition, blood PCR or LAMP can not conclusively prove the presence of live *Leishmania* parasites. Positive results may be due to parasite DNA circulating in the blood having leached out from infected skin or internal organs (Banu et al., 2016). Therefore, follow-up monitoring for clinical signs is crucial for validating the effectiveness of such sensitive methods for identification of asymptomatic cases and future prevention of disease.

Control of vector-borne diseases calls for the accurate appraisal of the hosts’ potential for infecting the arthropod vectors. Diagnosis of host infectiousness is optimally achieved by determining the infection rates of insectary-reared vectors that had fed on infected human volunteers (xenodiagnosis). Unfortunately, xenodiagnosis for leishmaniasis is an intricate operation encumbered by technical, logistical and ethical hurdles. We are currently working on replacement technologies based on skin microbiopsies that will be analyzed in the field using *Leishmania* specific LAMP to identify those persons that serve as reservoir hosts for propagating VL in the community and treat them.

## Figures and Tables

**Table 1:** DNA sequences of the primers designed for the three LAMP systems. Two targeting the ITS1 gene (See Fig. 1) and one targeting the *L. donovani*repeat DNA region.

LAMP System	Primer Name	Primer sequence
LITS-LAMP1	LITSF3.1	TTTCCCACATACACAGCAAA
	LITSB3.3	GCGGCGTGTTGTTTTTTG
	LITSFIP1	CGCCAAAAACCGAAACGCCTTTTCAGTAAAAAAAGGCCGATCG
	LITSBIP3	CGCAAACGGCGCATGGGAGAAGCTTTTCTGAGAATATGGCATGCACG
LITS-LAMP2	LITSF3.2	GTGGATAACGGCTCACATAA
	LITSB33	GCGGCGTGTTGTTTTTTG
	LITSFIP2	CTGCAATTGATACCACTTATCGCACTTTTACTTGGCTTCCTATTTCGTTG
	LITSBIP3	CGCAAACGGCGCATGGGAGAAGCTTTTCTGAGAATATGGCATGCACG
L151-LAMP	L151F3	GATGAGAAGCTCACGGAG
	L151B3	ATCCTCCTCCTCGTCTTC
	L151FIP	CGGGTACGTGAGTCCGTATTTTGTCTGCCAGTCAACAAGA
	L151BIP	GAAGCTCATGATCGAGAAGGAGTTTTTTCGTCTGATGCGTTGCT

**Table 2:** Parasite concentrations (per ml) in finger prick blood of volunteers from northern Ethiopia estimated by qRT-kDNA PCR. Results are compared with the corresponding results obtained by the different LAMP assays. Positive LAMP results are designated by the take-off time in mins (see materials and methods).

Sample number	History of VL	Parasite/ml qRT-kDNA-PCR	Estimated take-off time of LAMP amplification (min)		
			LITS- LAMP1	LITS- LAMP2	L151 LAMP
1	+	0	–	–	61
2	–	0	–	–	–
3	–	0	–	–	–
4	–	0	–	–	–
5	–	0	–	–	–
6	+	0	–	–	67
7	–	0	–	–	–
8	–	0	–	–	–
9	–	0	–	–	–
10	–	0	–	–	–
11	+	18	69	57	–
12	–	0	–	–	–
13	–	0	–	–	–
14	–	0	–	–	–
15	–	0	–	–	–
16	+	0	–	–	–
17	–	0	–	–	–
18	–	0	–	–	–
19	–	0	–	–	–
20	–	3	–	–	–
21	+	11	–	78	63
22	–	595	59	54	52
23	–	0	–	–	86
24	–	59	–	85	65
25	–	0	–	–	–
26	+	0	–	–	–
27	–	0	–	72	86
28	–	0	–	62	65
29	–	0	–	48	–
30	–	0	–	48	103
31	+	0	–	41	34
32	–	0	–	57	–
33	–	0	–	47	–
34	–	9	–	–	–
35	–	0	–	–	–
36	+	2	75	41	34
37	–	0	–	59	–
38	–	1	–	43	–
39	–	0	–	–	–
40	+	0	56	44	58
41	–	0	60	54	–
42	–	23	–	42	52
43	–	0	60	53	–
44	–	0	–	45	–

**Table 3:** A four by four matrix depicting the relationships between the different LAMP systems and the qRT-kDNA PCR results among the 44 individuals tested. Total numbers detected by each system are in the shaded cells. Note higher sensitivity of the LITS LAMP2 system compared with the PCR as well as the other two LAMP systems.
